# Supplementary material for: Transcriptome Analysis Reveals Vimentin-Induced Disruption of Cell–Cell Associations Augments Breast Cancer Cell Migration
Source: Cells. 2022 Dec 13;11(24):4035. doi: 10.3390/cells11244035 (PMC9776984; doi:10.3390/cells11244035)
Supplement: Supplementary file 1 [file cells-11-04035-s001.zip › DEGs and enrichment analysis.zip!/DEGs and enrichment analysis/WT_VIMvsVector KEGG/DOWN/WT_VIMvsVector_DOWN.pathway_map.zip!/WT_VIMvsVector_DOWN.pathway_map/src/hsa05167.html]

hsa05167


- hsa:23710

- Down regulated genes

ENSG00000139112(-1.85791559298273)

- hsa:23710

- Down regulated genes

ENSG00000139112(-1.85791559298273)

- hsa:3576

- Down regulated genes

ENSG00000169429(-3.18264312774765)

- hsa:2920

- Down regulated genes

ENSG00000081041(-2.33759639518744)

- hsa:1827

- Down regulated genes

ENSG00000159200(-2.06753401889746)

Close
